# Minimally Invasive Endoscopic and Surgical Management of Rectal Neoplasia

**DOI:** 10.3390/cancers14040948

**Published:** 2022-02-14

**Authors:** Sarah S. Al Ghamdi, Ira Leeds, Sandy Fang, Saowanee Ngamruengphong

**Affiliations:** 1 Division of Gastroenterology and Hepatology, Department of Medicine, King Abdulaziz University, Jeddah 21589, Saudi Arabia; ssalghamdi3@kau.edu.sa; 2 Division of Colon and Rectal Surgery, Department of Surgery, Yale School of Medicine, New Haven, CT 06510, USA; ira.leeds@yale.edu; 3 Department of Surgery, Johns Hopkins Hospital, Baltimore, MD 21224, USA; sfang7@jhmi.edu; 4 Division of Gastroenterology and Hepatology, Johns Hopkins Hospital, Baltimore, MD 21224, USA

**Keywords:** rectum, neoplasia, adenoma, submucosal invasion, cancer, polyp, endoscopic resection, minimally invasive, surgery

## Abstract

**Simple Summary:**

Rectal cancer accounts for one-third of colorectal cancer cases annually. It is preventable with early screening and appropriate management of precancerous lesions. Advancements in diagnostic and therapeutic strategies are ongoing and have contributed to improving patient outcomes. In this review, we summarize the available minimally invasive endoscopic and surgical management options of rectal neoplasia.

**Abstract:**

Rectal cancer demonstrates a characteristic natural history in which benign rectal neoplasia precedes malignancy. The worldwide burden of rectal cancer is significant, with rectal cancer accounting for one-third of colorectal cancer cases annually. The importance of early detection and successful management is essential in decreasing its clinical burden. Minimally invasive treatment of rectal neoplasia has evolved over the past several decades, which has led to reduced local recurrence rates and improved survival outcomes. The approach to diagnosis, staging, and selection of appropriate treatment modalities is a multidisciplinary effort combining interventional endoscopy, surgery, and radiology tools. This review examines the currently available minimally invasive endoscopic and surgical management options of rectal neoplasia.

## 1. Introduction

Colorectal cancer (CRC) is the third leading cause of cancer death worldwide, with rectal cancer accounting for 29% of new cases annually [1]. There is substantial evidence that CRC can be prevented by endoscopic detection and removal of adenomatous polyps [2]. While endoscopic resection is favored for benign lesions or early cancers, minimally invasive surgical techniques such as laparoscopic or robotic surgery tend to be preferred for advanced malignancies. However, there remains controversy as to what defines an estimated risk of lymph node (LN) metastasis in malignant lesions and the need for subsequent surgical therapy. This article will review the current literature pertaining to rectal neoplasia and the existing minimally invasive therapies.

Rectal cancer demonstrates a characteristic natural history. Rectal neoplasia comprises benign or malignant lesions, with benign neoplasia being a known precursor for most rectal cancers. Benign rectal polyps are neoplastic rectal lesions that have no histologic evidence of underlying malignancy. These encompass benign polyps with no malignant potential (e.g., hyperplastic) and neoplastic polyps with malignant potential (namely tubular, tubulovillous or villous adenomas). The latter may contain dysplastic changes (low- or high-grade dysplasia), which are considered noninvasive if they are located above the lamina propria. Malignant rectal polyps are lesions with malignant cells that have penetrated the muscularis mucosa and invaded the submucosa but have not extended into the muscularis propria [3]. These lesions are classified as pT1 in the 8th edition of the TNM classification system (AJCC staging system). A synonymous term for such lesions is submucosally invasive lesions/polyps. The relevance of submucosal invasion (SMI) lies in the fact that it coincides with possible lymphatic and/or vascular metastasis. Low-risk features of deep SMI include <1 mm (1000 mm) of SMI and absence of poor differentiation, lymphovascular invasion, and exclusion of tumor budding. When one of these criteria is not fulfilled, endoscopic resection might be inadequate, and surgical resection with lymph node dissection is recommended due to the associated high risk of residual cancer (specifically lymph node metastases) after endoscopic resection [4,5]. Although most polyps are benign, the incidence of malignant polyps increases with polyp size and has been reported as high as 40% in polyps ≥2 cm in size [6].

**Figure 1:**
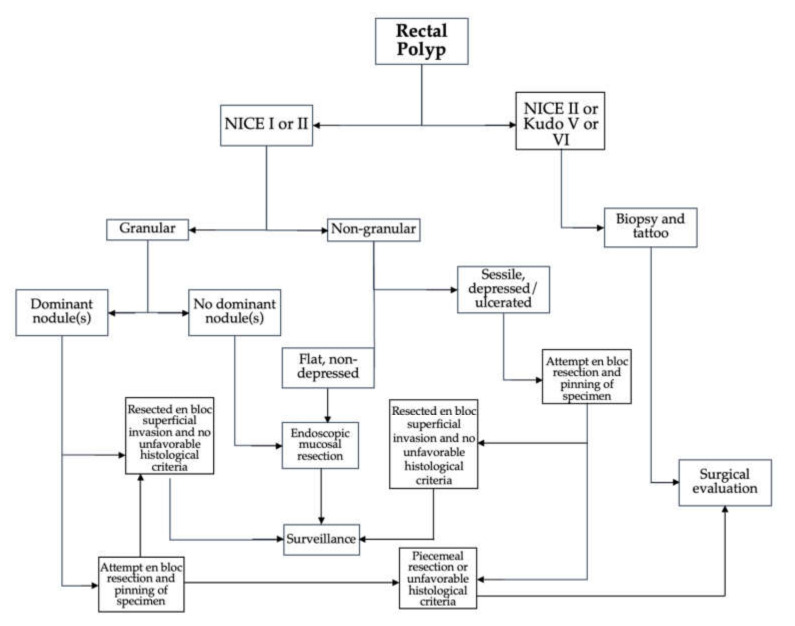
Algorithm for approach to rectal polyp assessment and management.

The therapeutic approach to rectal neoplasia is determined by several factors [3]. This includes lesion histology and anatomic stage, as determined macro- and microscopically, as well as radiologically. Size tends to be a more minute concern, as most large lesions are resectable endoscopically irrespective of size. The choice between an endoscopic or surgical approach may also differ based on resource availability, clinical expertise, and other factors. The clinical dilemma that is encountered by malignant polyps is whether surgical resection of the affected rectal segment is necessary following endoscopic resection. This is dependent on certain endoscopic and histologic features that aid in risk stratification. A modified version of the American Gastroenterological Association algorithm for the recognition and management of malignant polyps is seen in Figure 1. Certain endoscopic features are associated with a higher risk of superficial SMI. These include large polyp size (≥2 cm), depressed or sessile morphology in nongranular lateral spreading tumors (LST-NG), and discrete nodules in granular lateral spreading tumors (LST-G) (Table 1). To optimize histopathologic assessment, lesions with these features should be considered for en bloc endoscopic resection. If R0 resection is achieved, endoscopic surveillance is recommended. If there is evidence of deep SMI, risk of LN involvement is 10–18%, so surgical consultation is recommended [4]. US NCCN guidelines recommend that patients with rectal polyps containing invasive cancer removed endoscopically with a “fragmented specimen or margin(s) that cannot be assessed” undergo additional surgical resection due to risk of lymphovascular invasion [7]. When compared to surgery, ESD, being less invasive, was also found to have shorter procedure times and hospitalizations in retrospective comparisons with transanal resection (TAR) and transanal endoscopic microsurgery (TEM), with potentially lower recurrence rates [8]. More recently, a meta-analysis and systematic review included six retrospective studies that compared ESD with TEM or transanal minimally invasive surgery (TAMIS). It was concluded that ESD and surgical techniques do not differ in terms of local recurrence, en bloc and R0 resection rates, procedure duration, length of hospitalization, or adverse event (AE) rates [9]. However, the limited number of cohort studies available only provides low certainty of evidence. To date, there are no randomized controlled trials that compare ESD and surgical approaches.

## 2. Approach to Diagnosis and Staging

Early endoscopic detection of high-risk features for SMI is critical to help determine the best management strategy. This is attained through precise assessment of lesion morphology, surface architecture and vessel patterns, all of which require endoscopic expertise. Optical diagnostic techniques using blue-light imaging (BLI) such as narrow-band imaging (NBI) or image-enhanced endoscopy (i-SCAN) combined with high-definition white-light imaging (HD-WLE) are becoming standard on most modern endoscopes. These utilize reflective light of varying wavelengths to highlight the surface and microcapillary architecture of the mucosa. Dye-based chromoendoscopy (CE) using contrast dyes such as methylene blue (MB) or indigo carmine also plays a role in visual analysis. This involves staining the rectal mucosa and thus increasing the contrast between normal mucosa and neoplasia, which allows for more detailed detection of mucosal structural variation and surface and vessel patterns. Dye and blue-light imaging are therefore complementary techniques as they provide subtly different information on surface integrity.

**Figure 2:**
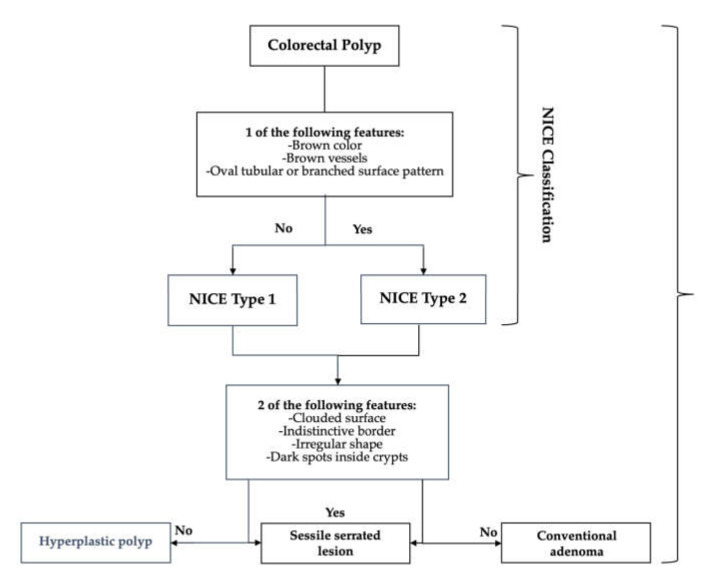
NBI International Colorectal Endoscopic (NICE) and (WASP) classifications for distinguishing between hyperplastic and adenomatous polyps.

Several endoscopic classifications have been validated. Kudo classification requires magnifying endoscopy to classify polyps based on their pit pattern according to their appearance, structure, and staining patterns. Type I and II are considered benign changes (normal, hyperplastic, or inflammatory polyps), and types III–V are considered neoplastic and malignant changes [10]. NBI International Colorectal Endoscopic (NICE) classification uses vascular patterns and surface patterns to distinguish between hyperplastic (Type 1) and adenomatous (Type 2) colon polyps. Type 3 represents features suggestive of deep SMI [11]. The WASP classification method (Figure 2) is an adjunct to the NICE classification which initially identifies type 1 lesions, followed by several sessile serrated lesion (SSL) features to differentiate between hyperplastic polyps and SSLs [11]. The BLI Adenoma Serrated International Classification (BASIC) system also classifies polyps into hyperplastic and adenomatous based on surface architecture, pit pattern, and vascular structure [12].

Features suggestive of deep SMI include a severely disrupted pit pattern with dye (Kudo classification types VN and VI) [10] and absent or irregularly thickened vessels using NBI (NICE classification type 3) [13]. Targeted biopsies from endoscopically suspicious areas are important for accurate diagnosis. That being said, the chance of false-negative biopsies in a lesion that harbors dysplasia or malignancy is not negligible, reaching up to 15.2% [14].

**Figure 3:**
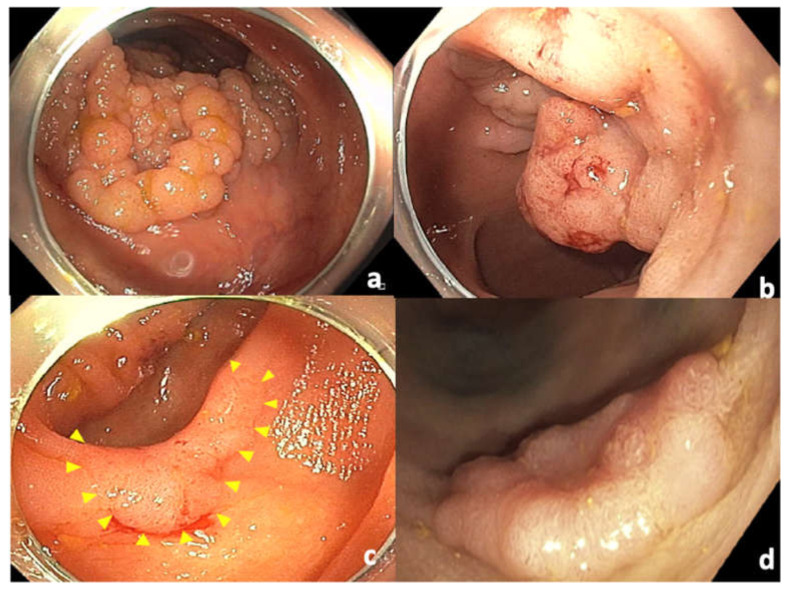
Classification of laterally spreading tumors. (**a**) Homogeneous granular laterally spreading tumor (LST-G); (**b**) nodular mixed type granular laterally spreading tumor (LST-MG); (**c**) flat nongranular laterally spreading tumor (LST-NG); (**d**) pseudodepressed nongranular laterally spreading tumor.

With regard to morphology, homogeneous LST-Gs (Figure 3a) are associated with a very low risk of SMI (0.5%), while nodular mixed LST-Gs (Figure 3b) have a higher risk of SMI (6%) in the presence of a dominant nodule. LST-NGs have a much higher risk of SMI (10.5% in NG flat elevated LSTs (Figure 3c) and 31.6% in NG pseudodepressed LSTs (Figure 3d)) [15]. Pseudodepressed LST-NG lesions are usually associated with multifocal invasion, the foci of which are often difficult to predict endoscopically [16]. In addition, these lesions are frequently associated with fibrosis, making EMR a less favorable option. Meanwhile, granular mixed LSTs (LST-GM) have an intermediate risk of covert SMI, leaving uncertainty as to the most appropriate endoscopic approach, specifically the need for en bloc resection [17,18]. Piecemeal resection of an LST-GM with unexpected covert SMI could lead to unnecessary surgery due to suboptimal histopathologic evaluation of degree of SMI and status of lateral margins [17]. This is especially relevant in the rectum, due to the negative impact of post-endoscopic surgery on postsurgical quality of life [19,20]. An analysis of data from 693 patients who underwent endoscopic resection (EMR or ESD) in patients with LST-GM found the risk of covert SMI to be approximately 10%. LST-GMs of 4 cm or more and a rectal location were found to be more high risk, and en bloc resection is therefore recommended [21].

In the presence of features suggestive of deep SMI, endoscopic resection should be avoided. This is because of the high risk for incomplete resection and LN metastasis. Instead, targeted biopsies from endoscopically suspicious areas should be performed, and the lesion should be marked distally with a tattoo to aid in detection at subsequent surgical resection [22]. In these situations, further evaluation for locoregional staging to detect lymph node involvement is also warranted. Endoscopic ultrasound (EUS) and pelvic MRI both have a well-established role in this regard. A meta-analysis concluded that improved diagnosis with EUS decreased the need for additional surgery and other associated problems from 24% to 5% [23]. Rectal EUS is superior to MRI at defining the depth of invasion of the muscularis mucosa and distinguishing T1 from T2 tumors (specificity 86% vs. 69%, *p* = 0.02) [24]. For T3 lesions, EUS was more sensitive (90% vs. 82%, *p* = 0.003). EUS also plays a significant role in early disease or smaller lesions that would not be seen on MRI, especially when local resection is contemplated. Although EUS was initially a mainstay for preoperative staging, advances in MRI have diminished this role. The NCCN clinical practice guidelines for rectal cancer suggest EUS for staging only if MRI is contraindicated [7]. MRI is preferred because EUS cannot identify the mesorectal fascia and in locally advanced disease fails to identify the circumferential resection margin (CRM), which are essential in determining the need for neoadjuvant chemotherapy [25]. MRI allows for adequate local staging (T stage and CRM involvement) and clear delineation of anatomic location with regard to sphincter involvement. Nodal staging is more challenging, as all modalities are equally insensitive in the range of 55% to 69% [26]. Although EUS and MRI can identify specific morphologic features of lymph nodes, these are often nondiagnostic. Lymph node size alone cannot be used to distinguish between benign reactive or malignant, as small lymph nodes may still harbor malignant cells [27]. Regardless of the modality selected, it is critical that it meets quality standards to provide precise conclusions.

## 3. Endoscopic Approaches to Therapy

### 3.1. Endoscopic Mucosal Resection

Endoscopic mucosal resection (EMR) can be considered for lesions that are definitely confined to the mucosal layer (Tis). EMR entails lifting the lesion by locally injecting a physiological saline or viscous solution into the underlying mucosa of the lesion. The lesion is then entrapped with a snare and resected with electrocautery. This can be done en bloc for lesions ≤2 cm in size or piecemeal for larger lesions to decrease the risk of deep mural injury (DMI). However, piecemeal resection is not without its shortcomings as it limits the ability to evaluate invasion depth and to determine a free margin. It is important to limit the number of resected specimens, as local recurrence rate increases with a greater number of resected pieces, so en bloc resection is typically preferred [28].

**Figure 4:**
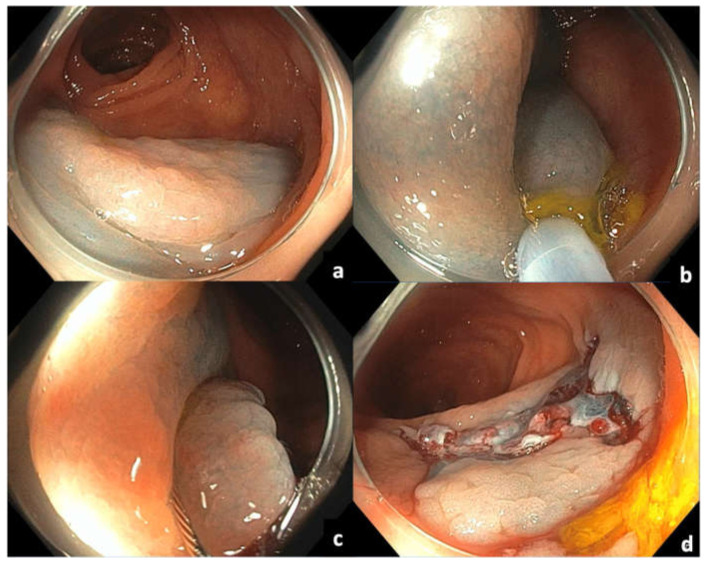
Cold EMR of sessile serrated ascending colon polyp: (**a**) Sessile serrated polyp in ascending colon following submucosal injection; (**b,c**) piecemeal resection with cold snare, taking into consideration adequate overlap of resection pieces and clear margins; (**d**) post-resection mucosal defect.

Several variations of EMR have come into favor, including cold EMR, which does not involve using electrocautery (Figure 4). There is growing evidence to suggest that cold EMR is safe and efficacious in removing polyps ≥10 mm in size, especially sessile serrated lesions (SSLs). An initial study found that the removal of SSLs could be performed effectively using cold EMR techniques, with a residual lesion rate of <1% [29]. A more recent retrospective study including 566 SSLs > 10 mm in size from 312 patients found the residual lesion rate to be 8% [30]. A meta-analysis that included 14 studies found that cold EMR for SSLs ≥10 mm was safe and had low residual polyp rates. Compared to hot EMR, cold EMR was associated with a lower rate of delayed bleeding, but there was no difference in residual polyp rate or other outcomes [31]. Cold snare polypectomy (CSP) without submucosal injection has also been used to resect large SSLs with a low recurrence rate. Several studies have also shown that piecemeal CSP is technically equally efficacious to EMR, with negligible recurrence in long-term follow-up. It has also been shown to be extremely safe, essentially eliminating the risk of post-EMR bleeding and deep mural injury (DMI) [32,33,34]. Due to the differences in techniques compared to hot EMR, it is suggested that cold snare resection be performed by using a dedicated cold snare while aiming for smaller pieces with attention to overlapping and obtaining wide healthy margins. Thermal ablation of the defect margin after EMR of large nonpedunculated colorectal polyps has been shown to significantly reduce residual or recurrent adenoma at first surveillance colonoscopy and is therefore recommended as adjunct therapy [35,36,37]. Another variant of cold EMR can also be performed underwater, known as underwater EMR (uEMR), which has the benefit of not requiring submucosal injection. Overall, EMR is a good option for most rectal polyps in the absence of extensive fibrosis or SMI, and for larger lesions in which en bloc resection is not deemed critical.

### 3.2. Endoscopic Full-Thickness Resection

Endoscopic full-thickness resection (eFTR) using a full-thickness resection device (FTRD) is a technique that permits deep resection of select lesions that are not amenable to conventional polypectomy or EMR. This approach entails performing circumferential markings of the lateral margins of the lesion with coagulation. A transparent cap with a 12.3 mm over-the-scope clip (OTSC) is then placed on a standard colonoscope. Once the lesion is reached, a grasping forceps is advanced through the working channel of the colonoscope to grasp the lesion and retracted into the cap until the lateral margins are visible in the cap. The OTSC is then deployed, isolating the target lesion to allow for resection. A preloaded polypectomy snare is then closed above the clip, and resection of the specimen is performed. Although FTRD has been found to be useful for fibrotic lesions either due to prior manipulation or SMI, the major limitation of this technique is that it cannot be used for lesions >3 cm in size [38].

Overall, the major AE rates reported with use of the FTRD are low. A multicenter prospective study that included patients from a large eFTR registry reported low overall AE rate (9.3% (*n* = 34/367)) for complex colorectal lesions [39].

### 3.3. Endoscopic Submucosal Dissection

**Figure 5:**
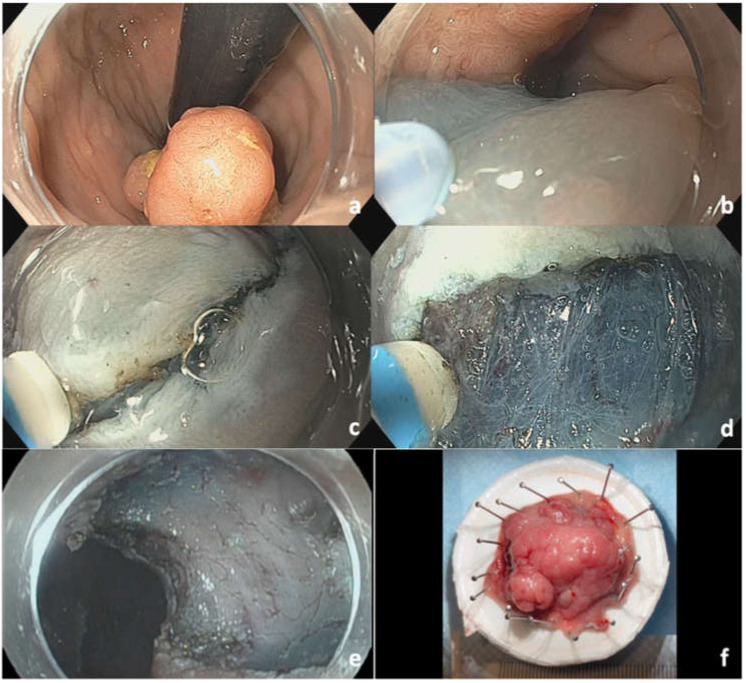
ESD of rectal polyp: (**a**) Rectal polyp seen in retroflexion; (**b**) mucosal injection with viscous solution and methylene blue; (**c**) mucosal incision using DualKnife J; (**d**) submucosal dissection; (**e**) resection site defect; (**f**) final 3 cm Paris classification Isp + IIa LST-mixed granular type.

Endoscopic submucosal dissection (ESD) has emerged in the past decade as an adjunct approach to EMR for management of colorectal lesions. An example of ESD of a rectal polyp is seen in Figure 5. The lesion is seen in retroflexion (Figure 5a). As with EMR, ESD also involves submucosal injection of a viscous solution (Figure 5b). In ESD, the surrounding circumferential normal mucosa of the lesion is initially incised using an ESD electrosurgical knife (Figure 5c). This is followed by submucosal dissection while expanding the submucosal space using a submucosal injection solution (Figure 5d), which allows for en bloc resection of lesions irrespective of size. Several variations of ESD have been described, and the Japan Gastroenterological Endoscopy Society guidelines provide specific terminology to distinguish each technique [40]. “Pure” or “conventional ESD” describes the technique in which submucosal dissection is completed with the electrosurgical knife without using a snare. “Precut EMR” involves making a circumferential incision at the outer margins of a lesion with a knife, followed by snare resection of the lesion without submucosal dissection. Conversely, “hybrid ESD” involves dissection of the submucosal layer with an electrosurgical knife or snare tip, followed by snare resection. The ultimate goal from all techniques is to achieve en bloc resection. Traction methods are often used during ESD and have been found to lead to shorter procedure times, improved R0 resection rates, and lower risk of perforation compared to conventional ESD [41]. These methods are constantly being adapted and involve the use of a combination of endoscopic clips, snares, sutures, and/or rubber bands [42]. Compared to EMR, ESD is more technically challenging and has a higher complication rate (perforation rate of 1.8% vs. 2.4%, OR = 0.56, *p* = 0.04) [43] and therefore requires dedicated training [44,45]. Although initially adopted in Asia long before it was introduced in North America, training opportunities and uptake of ESD in North American centers are increasing [46,47]. A recent large prospective study on ESD in North America found R0 resection rates to be 84.2% and 78.3% in colon and rectal ESD, which is close to target benchmarks achieved in Asian countries [48]. In this study bleeding and perforation were reported in 2.3% and 2.9% of the cases, respectively.

The primary advantage of ESD compared to EMR is a higher en bloc resection rate of large rectal lesions that would have been resected surgically prior to the introduction of ESD. ESD also has a lower recurrence rate compared to EMR (0.9–2% vs. 12.2–14%), which is dependent on the number of specimens removed during EMR, as well as histopathological features [49,50]. En bloc resection is particularly important for lesions in which SMI is suspected, as it provides precise prognostic histopathological information. ESD also plays a role for lesions with significant submucosal fibrosis, due to either prior tattooing or manipulation by partial resection or extensive biopsies. The American Society for Gastrointestinal Endoscopy also recommends en bloc resection by EMR or ESD of endoscopically visible dysplastic lesions in patients with inflammatory bowel disease (IBD) [51]. A recent meta-analysis including seven studies concluded that nonpolypoid dysplasia associated with IBD can be resected endoscopically with a low recurrence rate, especially by ESD [52].

## 4. Surgical Approaches to Therapy

The surgical approach to rectal cancer (be it local or radical excision), depends upon the clinical stage, size, and location of the primary tumor. A local excision is usually performed transanally, while radical excision is performed transabdominally with either a sphincter-sparing procedure such as low anterior resection or an abdominoperineal resection.

### 4.1. Transanal Approaches

The transanal approach affords the opportunity to avoid open surgery for very early rectal cancer with favorable features. Transanal minimally invasive surgery (TAMIS) is a technique developed to essentially perform single-port laparoscopic surgery through the open anal sphincter complex to locally excise low- to mid-rectal tumors. Although an early tumor can be fully excised with a TAMIS technique, the specimen is a simple full-thickness excision and thus lacks the TME component that has been shown to be such a major contributor to limiting local recurrence. Thus, a TAMIS approach employs specific selection criteria to ensure that only the lowest risk tumors for lymph node metastases are eligible. The primary tumor must be less than 3 cm in size, demonstrate well- or moderately differentiated histopathology without lymphovascular or perineural invasion, and have minimal submucosal invasion (e.g., sm1 or sm2 by Kikuchi classification) [7].

Long-term survival of surgically resectable rectal cancer is closely associated with local recurrence. Therefore, improvements to surgical techniques that are associated with reduced local recurrence can have a major impact on long-term outcomes. The greatest advance in our understanding of the surgical care of rectal cancer in the last thirty years is the understanding that equally important to a negative circumferential margin (i.e., “no ink on tumor”) is the en bloc prophylactic lymphadenectomy of the surrounding mesorectal fat packet known as a total mesorectal excision (TME). Widespread adoption of resection in the TME plane has led to serially reproduced local recurrence rates dropping from approximately 20% to less than 5% [53]. This technique remains the gold-standard rectal cancer care today and is applicable to both low anterior and abdominoperineal resections.

While the need for TME remains for most rectal cancer care, how one achieves a complete TME specimen continues to evolve. The anatomy of the pelvis is not always amenable to a meticulous TME plane dissection, and laparoscopic and robotic-assisted transabdominal techniques have been introduced to help overcome these difficulties. In addition, minimally invasive techniques are known to have faster recovery and less pain [54]. Noninferiority studies of these novel approaches have demonstrated mixed results. The quality of TME with a laparoscopic approach failed noninferiority to open surgery in both the Australasian Laparoscopic Cancer of the Rectum Randomized Clinical Trial (ALaCaRT) and the U.S.-based Alliance Z6051 trial [55]. However, early long-term results from both studies have shown no statistically significant difference in local recurrence [56]. Robotic-assisted techniques have not been shown to affect technical difficulty or short-term outcomes compared to conventional laparoscopy [57]. Another technique currently under investigation is the use of a combined transabdominal and transanal approach that facilitates laparoscopic or robotic-assisted transanal dissection of the mesorectal plan (taTME) to overcome the difficulties of transabdominal dissection. taTME largely remains investigational at this time with results expected from a major taTME series by 2024. In aggregate, these findings suggest that each approach may have a role in the surgeon’s armamentarium with appropriate selection based on surgeon experience and patient-specific factors.

### 4.2. Locally Advanced Rectal Cancer

Although treatment success (e.g., improved 5-year survival, reduced local recurrence) for rectal cancer has steadily improved over the last two decades, bulky, locally advanced tumors continue to be a challenge. With locally invasive tumors that have histopathologically favorable features (e.g., no violation of the mesorectal fascia, no invasion of the intersphincteric plane), recurrence rates are less than 2%. However, conventional multimodal therapy for T4 tumors extending beyond the mesorectal plane has recurrence rates greater than 20% [58].

Reducing the recurrence rate in locally advanced rectal cancer remains an active area of investigation. In these cases, the paradigm extends beyond total mesorectal excision to push the boundaries of resectability. There are three distinct scenarios of locally advanced disease that guide surgical decision-making. First, the best-studied scenario is when adjacent organs are secondarily involved in a large, bulky rectal primary. En bloc resection, or pelvic exenteration, of the rectum with the involved middle (vagina) and anterior (bladder) compartments of the pelvis confer a survival benefit for otherwise locally contained disease [59,60].

Second, a more recent consideration is whether lateral pelvic lymph nodes represent locally advanced or metastatic disease. The 8th edition of the American Joint Committee on Cancer regards lymph nodes along the internal and external iliac vessels as metastatic disease. However, evidence continues to mount that nodal involvement of the lateral pelvic lymph nodes has a prognosis more consistent with nonmetastatic mesorectal lymph node-positive disease, and thus should be staged and managed as locally advanced disease [61]. Based on this new perspective, lateral lymphadenectomy in addition to total mesorectal excision with neoadjuvant chemoradiotherapy is recommended when clinically positive nodal disease is present in these anatomic regions [7,62]. A future area of investigation remains whether prophylactic lateral lymphadenectomy may be indicated given the benefits demonstrated by total mesorectal excision, another form of prophylactic lymphadenectomy.

The final scenario of locally advanced cancer is a primary tumor that appears resectable on preoperative imaging but presents intraoperatively with a threatened margin of an unresectable adjacent structure. Given the considerations for adjacent organ and lymph node resection described above, the most common presentation of this scenario is a locally advanced rectal tumor with favorable imaging but intraoperatively is found to have dense fibrosis to the bony pelvis, and a microscopic disease-free plane cannot be fully appreciated—a threatened R1 resection. In these select cases, intraoperative radiation therapy (IORT) is an adjunctive therapeutic option that may be superior to conventional surgical resection and chemoradiation therapy alone for preventing local recurrence [63]. Guidelines emphasize that IORT is not appropriate for cases of gross disease involvement (R2) where further consideration of extreme resection or palliation would be more appropriate [7].

## 5. Future Directions

Ongoing refinement of endoscopic and surgical devices and techniques has significantly impacted the growth of the field. New traction devices to facilitate ESD have come into play, such as the through-the-scope Tracmotion device by Fujifilm (Lexington, MA, USA). Robotic manipulation devices such as the EndoMaster EASE System (EndoMaster Pte Ltd., Singapore) that can actively guide accessory instruments in various directions to allow for easier retraction and dissection are also being studied in animal models [64,65]. All of these advances aim to facilitate the performance and safety of ESD and increase utilization. Development of diagnostic imaging modalities to help distinguish benign from malignant tumors, assess tumor grade, delineate tumor extent, and define risk factors that may influence are also under development. These include functional and molecular imaging techniques such as diffusion-weighted MR, perfusion CT, and hybrid PET/CT imaging. Blood oxygenation level-dependent MRI and MR spectroscopy represent other advanced imaging modalities that are available but still underutilized, all of which may provide clinical advantages [66]. Emerging also are artificial intelligence systems involving real-time computer-aided detection (CADe) to aid detection of colorectal neoplasia by flagging suspected lesions with visual and acoustic notifications. Available evidence established that the incorporation of artificial intelligence results in a significant increase in detection of colorectal neoplasia, especially nonadvanced adenomas and polyps [67,68].

As novel diagnostic and therapeutic regimens evolve, the role of a multidisciplinary team (MDT) for risk stratification and defining optimal treatment plans becomes more critical. The goal of an MDT include providing recommendations based on the team consensus while integrating the complementary areas of expertise; reaching evidence-based recommendations based on national and international guidelines; ensuring effective communication for the coordination of care; educating team members of developments within specific areas of expertise; data collection and audit of outcomes; ongoing quality improvement in diagnosis, staging, treatment and surveillance; and encouraging participation in clinical trials. Multidisciplinary management should be implemented as the standard of care worldwide, with an aim to ensure that we work to continually improve quality and outcomes in rectal cancer [69].

## 6. Conclusions

Rectal neoplasia represents a substantial disease burden worldwide, with CRC being the third leading cause of cancer death worldwide and rectal cancer accounting for one-third of new cases annually [1]. Diagnosis and staging require a multimodal approach that involves endoscopy, EUS, and imaging modalities. Advancements in these strategies will aid more targeted therapies and allow for better risk stratification of patients who require further surgical management following endoscopic resection. Treatment plans require thorough MDT planning involving endoscopists, surgeons and radiologists to determine the most appropriate therapeutic approach. Generally, in the absence of features suggestive of deep SMI, endoscopic resection is predominantly feasible. Prospective studies comparing the currently available minimally invasive techniques are required to enhance implementation and improve outcomes.

## Figures and Tables

**Table 1:** Criteria for definition of high-risk and low-risk features for submucosal invasion (SMI).

High Risk	Low Risk
Poor differentiation	Well and moderate differentiation
>1 mm (1000 mm) of SMI	<1 mm (1000 mm) of SMI
Presence of tumor budding	Absence of tumor budding
Presence of lymphovascular invasion	Absence of lymphovascular invasion
Large polyp size (≥2 cm)	
Depressed or sessile morphology in nongranular lateral spreading tumors (LST-NG)	
Discrete nodules in granular lateral spreading tumors (LST-G)	

## References

[1] Siegel R.L., Miller K.D., Jemal A. (2020). Cancer statistics, 2020. CA Cancer J. Clin..

[2] Brenner H., Stock C., Hoffmeister M. (2014). Effect of screening sigmoidoscopy and screening colonoscopy on colorectal cancer incidence and mortality: Systematic review and meta-analysis of randomised controlled trials and observational studies. BMJ.

[3] Shaukat A., Kaltenbach T., Dominitz J.A., Robertson D.J., Anderson J.C., Cruise M., Burke C.A., Gupta S., Lieberman D., Syngal S. (2020). Endoscopic Recognition and Management Strategies for Malignant Colorectal Polyps: Recommendations of the US Multi-Society Task Force on Colorectal Cancer. Gastroenterology.

[4] Choi J.Y., Jung S.A., Shim K.N., Cho W., Keum B., Byeon J., Huh K., Jang B., Chang D., Jung H. (2015). Meta-analysis of predictive clinicopathologic factors for lymph node metastasis in patients with early colorectal carcinoma. J. Korean Med. Sci..

[5] Pimentel-Nunes P., Dinis-Ribeiro M., Ponchon T., Repici A., Vieth M., De Ceglie A., Amato A., Berr F., Bhandari P., Bialek A. (2015). Endoscopic submucosal dissection: European Society of Gastrointestinal Endoscopy (ESGE) Guideline. Endoscopy.

[6] Nakajima T., Saito Y., Tanaka S., Iishi H., Kudo S., Ikematsu H., Igarashi M., Saitoh Y., Inoue Y., Kobayashi K. (2013). Current status of endoscopic resection strategy for large, early colorectal neoplasia in Japan and Other Interventional Techniques. Surg. Endosc..

[7] National Comprehensive Cancer Network (2021). National Comprehensive Cancer Network Guidelines, Rectal Cancer; Version 2.2021. https://www.nccn.org/professionals/physician_gls/pdf/rectal.pdf.

[8] Kiriyama S., Saito Y., Matsuda T., Nakajima T., Mashimo Y., Joeng H., Moriya Y., Kuwano H. (2011). Comparing endoscopic submucosal dissection with transanal resection for non-invasive rectal tumor: A retrospective study. J. Gastroenterol. Hepatol..

[9] Sagae V.M.T., Ribeiro I.B., de Moura D.T.H., Brunaldi V., Logiudice F., Funari M., Baba E., Bernardo W., de Moura E. (2020). Endoscopic submucosal dissection versus transanal endoscopic surgery for the treatment of early rectal tumor: A systematic review and meta-analysis. Surg. Endosc..

[10] Kudo S., Hirota S., Nakajima T., Hosobe S., Kusaka H., Kobayashi T., Himori M., Yagyuu A. (1994). Colorectal Tumours and Pit Pattern. J. Clin. Pathol..

[11] Jspeert J.E., Bastiaansen B.A., van Leerdam M.E., Meijer G.A., van Eeden S., Sanduleanu S., Schoon E.J., Bisseling T.M., Spaander M.C., van Lelyveld N. (2016). Dutch Workgroup serrAted polypS & Polyposis (WASP). Development and validation of the WASP classification system for optical diagnosis of adenomas, hyperplastic polyps and sessile serrated adenomas/polyps. Gut.

[12] Bisschops R., Hassan C., Bhandari P., Coron E., Neumann H., Pech O., Correale L., Repici A. (2018). BASIC (BLI Adenoma Serrated International Classification) classification for colorectal polyp characterization with blue light imaging. Endoscopy.

[13] Hayashi N., Tanaka S., Hewett D.G., Kaltenbach T.R., Sano Y., Ponchon T., Saunders B.P., Rex D.K., Soetiko R.M. (2013). Endoscopic prediction of deep submucosal invasive carcinoma: Validation of the Narrow-Band Imaging International Colorectal Endoscopic (NICE) classification. Gastrointest. Endosc..

[14] Yang D., Aihara H., Perbtani Y.B., Wang A.Y., Aadam A.A., Tomizawa Y., Hwang J.H., Zou B., Natov N.S., Siegel A. (2019). Safety and efficacy of endoscopic submucosal dissection for rectal neoplasia: A multicenter North American experience. Endosc. Int. Open.

[15] Kudo S., Lambert R., Allen J.I., Fujii H., Fujii T., Kashida H., Matsuda T., Mori M., Saito H., Shimoda T. (2008). Nonpolypoid neoplastic lesions of the colorectal mucosa. Gastrointest. Endosc..

[16] Uraoka T., Saito Y., Matsuda T., Ikehara H., Gotoda T., Saito D., Fujii T. (2006). Endoscopic indications for endoscopic mucosal resection of laterally spreading tumours in the colorectum. Gut.

[17] Bogie R., Veldman M., Snijders L., Winkens B., Kaltenbach T., Masclee A., Matsuda T., Rondagh E., Soetikno R., Tanaka S. (2018). Endoscopic subtypes of colorectal laterally spreading tumors (LSTs) and the risk of submucosal invasion: A meta-analysis. Endoscopy.

[18] Fuccio L., Repici A., Hassan C., Ponchon T., Bhandari P., Jover R., Triantafyllou K., Mandolesi D., Frazzoni L., Bellisario C. (2018). Why attempt en bloc resection of non-pedunculated colorectal adenomas? A systematic review of the prevalence of superficial submucosal invasive cancer after endoscopic submucosal dissection. Gut.

[19] Rausa E., Kelly M.E., Bonavina L., O’Connell P.R., Winter D.C. (2017). A systematic review examining quality of life following pelvic exenteration for locally advanced and recurrent rectal cancer. Color. Dis..

[20] Wrenn S.M., Cepeda-Benito A., Ramos-Valadez D.I., Cataldo P.A. (2018). Patient perceptions and quality of life after colon and rectal surgery: What do patients really want?. Diseases of the Colon and Rectum.

[21] D’Amico F., Amato A., Iannone A., Trovato C., Romana C., Angeletti S., Maselli R., Radaelli F., Fiori G., Viale E. (2020). Risk of Covert Submucosal Cancer in Patients With Granular Mixed Laterally Spreading Tumors. Clin. Gastroenterol. Hepatol..

[22] Medina-Prado L., Hassan C., Dekker E., Bisschops R., Alfieri S., Bhandari P., Bourke M., Bravo R., Bustamante-Balen M., Dominitz J. (2021). When and How To Use Endoscopic Tattooing in the Colon: An International Delphi Agreement. Clin. Gastroenterol. Hepatol..

[23] Worrell S., Horvath K., Blakemore T., Flum D. (2004). Endorectal ultrasound detection of focal carcinoma within rectal adenomas. Am. J. Surg..

[24] Bipat S., Glas A.S., Slors F.J.M., Zwinderman A.H., Bossuyt P.M.M., Stoker J. (2004). Rectal cancer: Local staging and assessment of lymph node involvement with endoluminal US, CT, and MR imaging—A meta-analysis. Radiology.

[25] Balyasnikova S., Brown G. (2016). Optimal Imaging Strategies for Rectal Cancer Staging and Ongoing Management. Curr. Treat. Options Oncol..

[26] Lutz M.P., Zalcberg J.R., Glynne-Jones R., Ruers T., Ducreaux M., Arnold D., Aust D., Brown G., Bujko K., Cunninghan C. (2016). Second St. Gallen European Organisation for Research and Treatment of Cancer Gastrointestinal Cancer Conference: Consensus recommendations on controversial issues in the primary treatment of rectal cancer. Eur. J. Cancer.

[27] Wilkinson N. (2020). Management of Rectal Cancer. Surg. Clin. N. Am..

[28] Oka S., Tanaka S., Saito Y., Iishi H., Kudo S., Ikematsu H., Igarashi M., Saitoh Y., Inoue Y., Kobayashi K. (2015). Local recurrence after endoscopic resection for large colorectal neoplasia: A multicenter prospective study in Japan. Am. J. Gastroenterol..

[29] Tutticci N.J., Hewett D.G. (2018). Cold EMR of large sessile serrated polyps at colonoscopy (with video). Gastrointest. Endosc..

[30] Tate D., Awadie H., Bahin F., Desomer L., Lee R., Heitman S., Goodrick K., Bourke M. (2018). Wide-field piecemeal cold snare polypectomy of large sessile serrated polyps without a submucosal injection is safe. Endoscopy.

[31] Thoguluva Chandrasekar V., Spadaccini M., Aziz M., Maselli R., Hassan S., Fuccio L., Duvvuri A., Frazzoni L., Desai M., Fugazza A. (2019). Cold snare endoscopic resection of nonpedunculated colorectal polyps larger than 10 mm: A systematic review and pooled-analysis. Gastrointest. Endosc..

[32] Van Hattem W., Shahidi N., Vosko S., Hartley I., Britto K., Sidhu M., Bar-Yishay I., Schoeman S., Tate D., Byth K. (2020). Piecemeal cold snare polypectomy versus conventional endoscopic mucosal resection for large sessile serrated lesions: A retrospective comparison across two successive periods. Gut.

[33] Kimoto Y., Sakai E., Inamoto R., Kurebayashi M., Takayanagi S., Hirata T., Suzuki Y., Ishii R., Konishi T., Kanda K. (2020). Safety and Efficacy of Cold Snare Polypectomy Without Submucosal Injection for Large Sessile Serrated Lesions: A Prospective Study. Clin. Gastroenterol. Hepatol..

[34] Mcwhinney C.D., Vemulapalli K.C., El Rahyel A., Abdullah N., Rex D.K. (2021). Adverse events and residual lesion rate after cold endoscopic mucosal resection of serrated lesions. Gastrointest. Endosc..

[35] Klein A., Tate D.J., Jayasekeran V., Hourigan L., Singh R., Brown G., Bahin F.F., Burgess N., Williams S.J., Lee E. (2019). Thermal Ablation of Mucosal Defect Margins Reduces Adenoma Recurrence After Colonic Endoscopic Mucosal Resection. Gastroenterology.

[36] Sidhu M., Shahidi N., Gupta S., Desomer L., Vosko S., Arnout van Hattem W., Hourigan L.F., Lee E.Y.T., Moss A., Raftopoulos S. (2021). Outcomes of Thermal Ablation of the Mucosal Defect Margin After Endoscopic Mucosal Resection: A Prospective, International, Multicenter Trial of 1000 Large Nonpedunculated Colorectal Polyps. Gastroenterology.

[37] Chandan S., Facciorusso A., Ramai D., Deliwala S., Mohan B.P., Kassab L.L., Draganov P.V., Othman M.O., Kochhar G.S. (2022). Snare tip soft coagulation (STSC) after endoscopic mucosal resection (EMR) of large (>20 mm) non pedunculated colorectal polyps: A systematic review and meta-analysis. Endosc. Int. Open.

[38] Trindade A., Kumta N., Bhutani M., Chandrasekhara V., Jirapinyo P., Krishnan K., Melson J., Pannala R., Parsi M., Schulman A. (2020). Devices and techniques for endoscopic treatment of residual and fibrotic colorectal polyps (with videos). Gastrointest. Endosc..

[39] Zwager L., Bastiaansen B., Bronzwaer M., Van Der Spek B., Heine G., Haasnoot K., Van Der Sluis H., Perk L., Boonstra J., Rietdijk S. (2020). Endoscopic full-thickness resection (eFTR) of colorectal lesions: Results from the Dutch colorectal eFTR registry. Endoscopy.

[40] Tanaka S., Kashida H., Saito Y., Yahagi N., Yamano H., Saito S., Hisabe T., Yao T., Watanabe M., Yoshida M. (2020). Japan Gastroenterological Endoscopy Society guidelines for colorectal endoscopic submucosal dissection/endoscopic mucosal resection. Dig. Endosc..

[41] Lopimpisuth C., Simons M., Akshintala V.S., Prasongdee K., Nanavati J., Ngamruengphong S. (2021). Traction-assisted endoscopic submucosal dissection reduces procedure time and risk of serious adverse events: A systematic review and meta-analysis. Surg. Endosc..

[42] Tsuji K., Yoshida N., Nakanishi H., Takemura K., Yamada S., Doyama H. (2016). Recent traction methods for endoscopic submucosal Dissection. World J. Gastroenterol..

[43] Zhao H.J., Yin J., Ji C.Y., Wang X., Wang N. (2020). Endoscopic mucosal resection versus endoscopic submucosal dissection for colorectal laterally spreading tumors: A meta-analysis. Rev. Esp. Enferm. Dig..

[44] Wang J., Zhang X.H., Ge J., Yang C.M., Liu J.Y., Zhao S.L. (2014). Endoscopic submucosal dissection vs endoscopic mucosal resection for colorectal tumors: A meta-analysis. World J. Gastroenterol..

[45] Kobayashi N., Yoshitake N., Hirahara Y., Konishi J., Saito Y., Matsuda T., Ishikawa T., Sekiguchi R., Fujimori T. (2012). Matched case-control study comparing endoscopic submucosal dissection and endoscopic mucosal resection for colorectal tumors. J. Gastroenterol. Hepatol..

[46] Kotzev A., Yang D., Draganov P. (2019). How to master endoscopic submucosal dissection in the USA. Dig. Endosc..

[47] McCarty T.R., Aihara H. (2020). Current state of education and training for endoscopic submucosal dissection: Translating strategy and success to the USA. Dig. Endosc..

[48] Draganov P., Aihara H., Karasik M., Ngamruengphong S., Aadam A., Othman M., Sharma N., Grimm I., Rostom A., Elmunzer B. (2021). Endoscopic Submucosal Dissection in North America: A Large Prospective Multicenter Study. Gastroenterology.

[49] Saito Y., Fukuzawa M., Matsuda T., Fukunaga S., Sakamoto T., Uraoka T., Nakajima T., Ikehara H., Fu K., Itoi T. (2010). Clinical outcome of endoscopic submucosal dissection versus endoscopic mucosal resection of large colorectal tumors as determined by curative resection. Surg. Endosc..

[50] Fujiya M., Tanaka K., Dokoshi T., Tominaga M., Ueno N., Inaba Y., Ito T., Moriichi K., Kohgo Y., Asahikawa P. (2015). Efficacy and adverse events of EMR and endoscopic submucosal dissection for the treatment of colon neoplasms: A meta-analysis of studies comparing EMR and endoscopic submucosal dissection. Gastrointest. Endosc..

[51] Shergill A., Lightdale M., Bruining D., Acosta R., Chandrasekhara V., Chatadi K., Anton Decker G., Early D., Evans J., Fanelli R. (2015). The role of endoscopy in inflammatory bowel disease. Gastrointest. Endosc..

[52] Chen W., Zhang Y.L., Zhao Y., Yang A.M., Qian J.M., Wu D. (2021). Endoscopic resection for non-polypoid dysplasia in inflammatory bowel disease: A systematic review and meta-analysis. Surg. Endosc..

[53] Quirke P., Steele R., Monson J., Grieve R., Khanna S., Couture J., O’Callaghan C., Myint A., Bessell E., Thompson L. (2009). Effect of the plane of surgery achieved on local recurrence in patients with operable rectal cancer: A prospective study using data from the MRC CR07 and NCIC-CTG CO16 randomised clinical trial. Lancet.

[54] Guillou P., Quirke P., Thorpe H., Walker J., Jayne D., Smith A., Heath R., Brown J. (2005). Short-term endpoints of conventional versus laparoscopic-assisted surgery in patients with colorectal cancer (MRC CLASICC trial): Multicentre, randomised controlled trial. Lancet.

[55] Fleshman J., Branda M., Sargent D., Boller A., George V., Abbas M., Peters W., Maun D., Chang G., Herline A. (2015). Effect of laparoscopic-assisted resection vs open resection of stage II or III rectal cancer on pathologic outcomes the ACOSOG Z6051 randomized clinical trial. JAMA J. Am. Med. Assoc..

[56] Stevenson A., Solomon M., Lumley J., Hewett P., Clouston A., Gebski V., Davies L., Wilson K., Hague W., Simes J. (2015). Effect of laparoscopic-assisted resection vs open resection on pathological outcomes in rectal cancer: The ALaCaRT randomized clinical trial. JAMA J. Am. Med. Assoc..

[57] Jayne D., Pigazzi A., Marshall H., Croft J., Corrigan N., Copeland J., Quirke P., West N., Rautio T., Thomassen N. (2017). Effect of robotic-assisted vs conventional laparoscopic surgery on risk of conversion to open laparotomy among patients undergoing resection for rectal cancer the rolarr randomized clinical trial. JAMA J. Am. Med. Assoc..

[58] Taylor F., Quirke P., Heald R., Moran B., Blomqvist L., Swift I., Sebag-Montefiore D., Tekkis P., Brown G. (2011). Preoperative high-resolution magnetic resonance imaging can identify good prognosis stage I, II, and III rectal cancer best managed by surgery alone: A prospective, multicenter, European study. Ann. Surg..

[59] Law W.L., Chu K.W., Choi H.K. (2000). Total pelvic exenteration for locally advanced rectal cancer. J. Am. Coll. Surg..

[60] PelvEx Collaborative (2019). Surgical and Survival Outcomes Following Pelvic Exenteration for Locally Advanced Primary Rectal Cancer: Results From an International Collaboration. Ann. Surg..

[61] Akiyoshi T., Watanabe T., Miyata S., Kotake K., Muto T., Sugihara K. (2012). Results of a Japanese nationwide multi-institutional study on lateral pelvic lymph node metastasis in low rectal cancer: Is it regional or distant disease?. Ann. Surg..

[62] Peacock O., Chang G.J. (2020). The Landmark Series: Management of Lateral Lymph Nodes in Locally Advanced Rectal Cancer. Ann. Surg. Oncol..

[63] Kusters M., Valentini V., Calvo F.A., Krempien R., Nieuwenhujizen G., Martijn H., Doglietto G., del Valle E., Roeder F., Buchler M. (2009). Results of European pooled analysis of IORT-containing multimodality treatment for locally advanced rectal cancer: Adjuvant chemotherapy prevents local recurrence rather than distant metastases. Ann. Oncol..

[64] Hwang M., Lee S.W., Park K.C., Sul H.J., Kwon D.S. (2020). Evaluation of a robotic arm-assisted endoscope to facilitate endoscopic submucosal dissection (with video). Gastrointest. Endosc..

[65] Chiu P.W.Y., Ho K.Y., Phee S.J. (2021). Colonic endoscopic submucosal dissection using a novel robotic system (with video). Gastrointest. Endosc..

[66] Mainenti P.P., Stanzione A., Guarino S., Romeo V., Ugga L., Romano F., Storto G., Maurea S., Brunetti A. (2019). Colorectal cancer: Parametric evaluation of morphological, functional and molecular tomographic imaging. World J. Gastroenterol..

[67] Hassan C., Spadaccini M., Iannone A., Maselli R., Jovani M., Chandrasekar V.T., Antonelli G., Yu H., Areia M., Dinis-Ribeiro M. (2021). Performance of artificial intelligence in colonoscopy for adenoma and polyp detection: A systematic review and meta-analysis. Gastrointest. Endosc..

[68] Barua I., Vinsard D.G., Jodal H.C., Løberg M., Kalager M., Holme Ø., Misawa M., Bretthauer M., Mori Y. (2021). Artificial intelligence for polyp detection during colonoscopy: A systematic review and meta-analysis. Endoscopy.

[69] Keller D.S., Berho M., Perez R.O., Wexner S.D., Chand M. (2020). The multidisciplinary management of rectal cancer. Nat. Rev. Gastroenterol. Hepatol..

